# Visualizing 3D Embryo and Tissue Morphology—A Decade of Using High-Resolution Episcopic Microscopy (HREM) in Biomedical Imaging

**DOI:** 10.3390/biomedicines10051123

**Published:** 2022-05-12

**Authors:** Stefan H. Geyer, Wolfgang J. Weninger

**Affiliations:** Division of Anatomy, Center for Anatomy and Cell Biology, Medical Imaging Cluster, Medical University of Vienna, Waehringer Str. 13, 1090 Vienna, Austria; stefan.geyer@meduniwien.ac.at

High-resolution episcopic microscopy (HREM) [1] is a straightforward method for generating digital volume data of organic tissue samples sized between hundreds of cubic microns and a few cubic centimeters. The data fit for immediate three-dimensional (3D) computer visualization and spatial analysis of tissue architecture and gene expression topology. Although specimen preparation and data generation are based on quite simple principles, the method is still constantly optimized and new scientific applications are explored.

HREM already proved to fit for visualizing embryo anatomy and the tissue architecture of samples and biopsies harvested from all sorts of animals, humans, plants, as well as synthetic skin substitutes, coated and uncoated paper, and many other materials [2]. The HREM workflow involves harvesting, fixation, dehydration, and resin embedding, followed by sectioning of the resin blocks on a special microtome. During sectioning, images of all freshly exposed block faces are scanned to produce a virtual stack of several thousands of perfectly aligned digital images [3,4,5]. The quality of the single images is close to the quality of digital images captured in a light-microscopy setting. Numeric resolution of the full 3D data is down to less than 1 cubic micron.

The collection of papers published in this Special Issue will provide and discuss examples for utilizing HREM and introduce new technical developments and data interpretation strategies.

Mark et al. [6] for example, study pathogenesis of anorectal malformations in a retinoic acid receptor (RAR) knock out mouse line. They employ HREM for screening the phenotype of mouse embryos from E10.5 to E15.5 after silencing RAR-coding genes at E10.5 and E11.5. Thanks to their holistic application of HREM, they detected a wide array of malformations, including the expected anorectal agenesis as well as malformations of the urogenital and cardiovascular system and ocular and nasal defects. They demonstrated that abnormalities of the umbilical arteries precede cloacal defects and concluded that the investigated RAR pathway plays a significant role in the development of the umbilical arteries and its derivatives. In summary, by relying on HREM imaging, the authors nicely demonstrate the effects defective RAR signaling pathways have on morphogenesis. The study thus emphasizes the usefulness of HREM for researching the mechanisms underlying morphogenesis and malformations.

Wendling et al. [7] demonstrate the versatility of their HREM-apparatus and the usefulness of HREM for characterizing tissues and organs of embryonic, but also adult mice. They visualize the developing nervous and urogenital system of embryos and examine the knee joint with ligaments as well as atherosclerotic plaques in the aorta of adult mice. Their study relies on surface rendered models after segmentation of the studied structures and demonstrates that HREM data fit for quick volumetric analysis. Furthermore, the results underline the importance of volumetry for understanding the growth and development of biomedical model organisms and demonstrate the importance of HREM data for the exact characterization of pathologies, such as atherosclerotic plaques in the adult mouse model.

The study of Pokhrel et al. [8], demonstrates that HREM is not limited to visualizing mice. They work with the chick and study the role the cell cycle regulator WEE1 plays in the diapause. Diapause refers to a developmental arrest that embryos of the blastoderm stage enter, if the temperature of the environment decreases below 21 °C. The authors show that WEE1 is upregulated at 12 °C and triggers an arrest of the cell cycle in the G2/M phase, while at 18 °C, lower WEE1 levels cause prolonged cell proliferation and lower survival rate. This was elegantly confirmed by exploring volume rendered computer models and quantifications of blastodermal components based on HREM data.

Technical issues are addressed by Reissig et al. [9]. The authors systematically characterize artefacts inherent to HREM data, by making use of more than 600 HREM data sets of E14.5 mouse embryos, produced under standardized conditions in the scope of the “Deciphering the mechanisms of developmental diseases” (DMDD) program. They characterize and categorize the artefacts and provide information about their frequency. In addition, they discuss the influence artefacts have on the correct interpretation of the phenotype of mouse embryos and provide examples for pitfalls in diagnosing malformations. Hence, the results prepare scientists for working with HREM data and assist them in their interpretation. Most importantly, they will form the basis for triggering efforts to optimize specimen processing and data generation protocols in order to overcome or at least minimize artefacts obscuring tissue information in HREM data.

Finally, Keuenhof et al. [10] address the role of HREM in novel, cutting-edge imaging approaches. They provide examples of multimodal imaging pipelines, in which HREM plays a central role. Such pipelines allow for multiscale 3D visualization of specimens, through their subsequent imaging with multiple imaging methods. Imaging pipelines thus provide the opportunity for a holistic visualization of organisms and materials at all levels of resolution. Subcellular structures and functional information are visualized in the context of cells, tissues, organs, and whole organisms. This sounds trivial, but a large number of obstacles, chiefly caused by different specimen processing requirements, have to be resolved. HREM proved to be combinable with quite a large number of different imaging modalities, including (micro) magnetic resonance tomography/imaging (MR), (micro) computed tomography (CT), optical coherence tomography (OCT), photo acoustic tomography (PAT), histopathology, and others. The paper thus nicely demonstrates the opportunities HREM offers in the scope of cross scale imaging.

In sum, this Special Issue represents a nice compendium of excellent research papers, which cover all major aspects of HREM imaging. It therefore represents a useful resource for exploring and using HREM in science, research, and teaching.
